# Low-Polarization, Broad-Spectrum Semiconductor Optical Amplifiers

**DOI:** 10.3390/nano14110969

**Published:** 2024-06-02

**Authors:** Meng Zhang, Tianyi Zhang, Hui Tang, Lei Liang, Yongyi Chen, Li Qin, Yue Song, Yuxin Lei, Peng Jia, Yubing Wang, Cheng Qiu, Yuntao Cao, Yongqiang Ning, Lijun Wang

**Affiliations:** 1 State Key Laboratory of Luminescence and Applications, Changchun Institute of Optics, Fine Mechanics and Physics, Chinese Academy of Sciences, Changchun 130033, China; zhangmeng223@mails.ucas.ac.cn (M.Z.); 18943085572@163.com (T.Z.); tanghui21@mails.ucas.ac.cn (H.T.); qinl@ciomp.ac.cn (L.Q.); songyue@ciomp.ac.cn (Y.S.); leiyuxin@ciomp.ac.cn (Y.L.); jiapeng@ciomp.ac.cn (P.J.); wangyubing@ciomp.ac.cn (Y.W.); qiucheng@ciomp.ac.cn (C.Q.); ningyq@ciomp.ac.cn (Y.N.); wanglj@ciomp.ac.cn (L.W.); 2 Daheng College, University of Chinese Academy of Sciences, Beijing 100049, China; 3 Jilin Changguang Jixin Technology Co., Ltd., No. 206, Software Road, Changchun 130022, China; 4 Jlight Semiconductor Technology Co., Ltd., No. 1588, Changde Road, Changchun 130102, China; 5 National Key Laboratory of Advanced Vehicle Integration and Control, China FAW Corporation Limited, Changchun 130000, China; caoyuntao@faw.com.cn

**Keywords:** semiconductor optical amplifier, gain sensitivity, polarization sensitivity, gain bandwidth, quaternary compound

## Abstract

Polarization-insensitive semiconductor optical amplifiers (SOAs) in all-optical networks can improve the signal-light quality and transmission rate. Herein, to reduce the gain sensitivity to polarization, a multi-quantum-well SOA in the 1550 nm band is designed, simulated, and developed. The active region mainly comprises the quaternary compound InGaAlAs, as differences in the potential barriers and wells of the components cause lattice mismatch. Consequently, a strained quantum well is generated, providing the SOA with gain insensitivity to the polarization state of light. In simulations, the SOA with ridge widths of 4 µm, 5 µm, and 6 µm is investigated. A 3 dB gain bandwidth of >140 nm is achieved with a 4 µm ridge width, whereas a 6 µm ridge width provides more output power and gain. The saturated output power is 150 mW (21.76 dB gain) at an input power of 0 dBm but increases to 233 mW (13.67 dB gain) at an input power of 10 dBm. The polarization sensitivity is <3 dBm at −20 dBm. This design, which achieves low polarization sensitivity, a wide gain bandwidth, and high gain, will be applicable in a wide range of fields following further optimization.

## 1. Introduction

In recent years, the demand for network traffic in new applications and businesses, such as cloud computing, the Internet of Things (IoT), high-definition video, and industrial interconnection, has surged [1,2]. According to publicly available data from the Ministry of Industry and Information Technology of China, the cumulative mobile internet traffic in China reached 3015 billion GB in 2023, a 228-fold increase over the past decade. Future applications of 6G networks will further focus on 3D immersive experiences, including augmented reality, smart interactions, holographic communication, digital twins, and other new applications, which pose significant challenges to the transmission capacity and speed of optical fiber communication systems, accelerating the upgrade of optical networks [3,4,5].

The C-band and the O-band are indeed the predominant frequency bands in optical communication systems. However, selective frequency fading due to chromatic dispersion significantly limits the transmission distance of high-speed digital links, driving the need for high-performance optical amplifiers [6]. Moreover, coherent light enables linear frequency conversion of the entire optical signal into a baseband electrical signal, better fulfilling the bandwidth requirements of optical networks [7]. Thus, coherent optical communication technology, integrating high-order modulation and digital coherent detection, has emerged as the preferred solution for long-distance, high-capacity, and high-speed optical communication. Core devices such as optical amplifiers must exhibit polarization-insensitive characteristics to accommodate more information and expand parallel channel counts beyond the C+L band. Current developments suggest that their transmission capacity could increase by up to five times [8]. Furthermore, they must evolve towards higher power levels to meet the demands of long-distance communication effectively [9,10,11,12].

Traditional methods involve the use of erbium-doped fiber amplifiers (EDFAs) for relay amplification. While EDFAs offer high gains and low noise figures, they have a narrow gain bandwidth of typically only about 60 nm and are bulky with high power consumption. Semiconductor optical amplifiers (SOAs) have significant advantages in miniaturization, energy efficiency, increased output power, reduced noise figure, and photonic integration [13,14,15,16,17]. Unlike polarization-sensitive SOAs, polarization-insensitive SOAs can amplify both TE- and TM-mode optical signals simultaneously, thereby enhancing the flexibility and scalability of networks. Therefore, polarization-insensitive SOAs are poised to become the core devices for future long-distance, ultra-high-speed coherent optical communication [18,19,20].

Several methods have been used to achieve polarization-state-insensitive SOAs. For example, two SOAs can be connected in series or in parallel using a combined gain compensation structure. The signal light is split into transverse electric (TE) and transverse magnetic (TM) polarized light using a polarizing beam splitter. The first SOA amplifies the TE polarized light, which is then transformed into TM polarized light by passing through a half-wavelength slice. Subsequently, the TM polarized light is converted to TE polarized light by passing through a half-wavelength slice. Finally, the amplified TM polarized light is combined with another amplified beam using a polarizing beam splitter (combined output) [21,22,23]. However, this method has several disadvantages, including coupling difficulties, a large size, high costs, and unstable external combinations. Alternatively, a polarization-insensitive SOA can be achieved using polarization-dependent loss. In this method, optical signals from the SOA output are coupled into a polarization-dependent loss unit, where the signal light undergoes a polarization-dependent gain, thus providing the SOA with polarization insensitivity [24]. However, the polarization-dependent loss unit has a predetermined optical fiber length, which incurs additional losses and complicates integration with other devices. Polarization-insensitive SOAs can also be fabricated by designing appropriate SOA waveguide structures and tuning the optical-field-limiting factors of the TE and TM modes. However, this approach requires submicron-dimensional accuracy in the active region, places high demands on the etching process, and is difficult to integrate with passive waveguides [25].

Notably, polarization-insensitive SOAs can be obtained by introducing varying degrees of strain within quantum wells or barrier layers through energy band engineering. Advantageously, this method offers high stability and easy integration [19,26,27,28]. Currently, the primary strain structures for achieving polarization-insensitive SOAs in multiple quantum wells (MQWs) are strain-compensated, trap-compressed, and barrier-stretched MQW structures [19]. The stretch–strain MQW system based on InGaAsP/InP has been reported to have a bandwidth of up to 30 nm at a wavelength of 1300 nm, a chip gain of approximately 15 dB, and a polarization sensitivity of less than 1.5 dB [29]. In WDM-PONs, an SOA with a greater ability to expand the bandwidth will increase the signal transmission capacity. Furthermore, asymmetric MQW (AMQW) structures have demonstrated greater spectral broadening capabilities than symmetric MQW structures [30]. At input powers of −10 dBm and −20 dBm, an SOA with an AMQW structure achieved a maximum gain of 20 dB at 1360 nm with a polarization sensitivity of less than 3 dB [31].

The SOA serves not only as a device sensitive to polarization but also imposes stringent requirements on high-performance parameters, including output power, bandwidth, and noise figure [32]. Although previous endeavors have made strides in partially refining the polarization characteristics of the SOA, the metrics related to output power and gain bandwidth have not witnessed substantial improvement or enhancement.

Therefore, this paper introduces a novel SOA device with high power, low polarization sensitivity, and a wide gain spectrum. Through meticulous optimization of the strain characteristics within the active region to minimize material mode gain discrepancies and enhance gain bandwidth, coupled with the optimization of the relationship between the optical confinement factor of the active region and the ridge width to achieve an asymmetric large cavity, the device’s saturated output power is significantly elevated. Additionally, the incorporation of tilted waveguides and wideband ultra-low reflectivity cavity facet coatings facilitates low-noise output. Consequently, the SOA attains notable performance benchmarks, including a maximum output power of 233 mW, a maximum gain of 32.89 dB, and a 3 dB gain bandwidth exceeding 140 nm.

In this investigation, a polarization-insensitive SOA featuring an InGaAlAs active region was proposed. The designed structure was subjected to simulation and fabrication, with a comprehensive analysis conducted on the impact of ridge width on device performance. Compared to previous methodologies, this design maintains relatively low polarization sensitivity even under small signal inputs, while also exhibiting a broader gain bandwidth, a higher gain, and saturated output power. These attributes have promising implications across a wide spectrum of applications.

## 2. Results and Discussion

### 2.1. Simulation and Design of the SOA Structure

**Figure 1:**
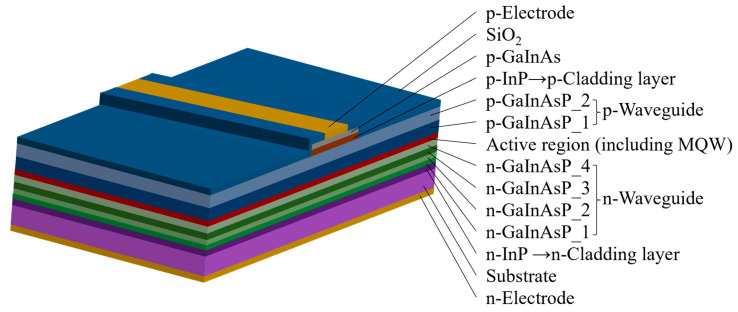
Epitaxial structure of the designed semiconductor optical amplifier (SOA).

Figure 1 shows the epitaxial structure of a polarization-insensitive SOA with high saturation output power. The active region comprises five strained quantum wells with thicknesses of 10 nm and six barrier layers with thicknesses of 12 nm, all of which consist of the quaternary compound InGaAlAs. A gradual waveguide structure is formed using two waveguide layers with different components and thicknesses on each side of the active region.

Specifically, the introduction of different degrees of doping improves the carrier injection efficiency and enhances carrier confinement. The core layer is flanked by n- and p-type doped cladding layers, which both consist of InP. The cladding layers are thicker than the core layer to improve the stability of the structure and enhance the light transmission efficiency in the active layer [33]. By varying the Ga and Al components in InGaAlAs, the refractive index of the material can be gradually decreased from the active layer toward each side, thereby confining the optical field to the active region and waveguide layer. High residual reflectivity at the end face can lead to an unstable optical output power [34] and increased noise. Therefore, an ideal SOA should have an end-face residual reflectivity of 0, but this is difficult to achieve in practice. Typically, a transmittance-enhancement film is applied to both ends of the SOA chip to reduce the end-face residual reflectance to less than 0.01%. Transparent windows and tilted waveguides can also be used to decrease the residual reflectance. In this study, we designed a waveguide structure with a tilt angle of 7°, as illustrated in Figure 1. To study the effect of ridge width on device performance, three chip structures with different ridge widths (4 µm, 5 µm, and 6 µm) were designed. The chip cavity had a length of 2500 µm and a width of 20 µm.

**Figure 2:**
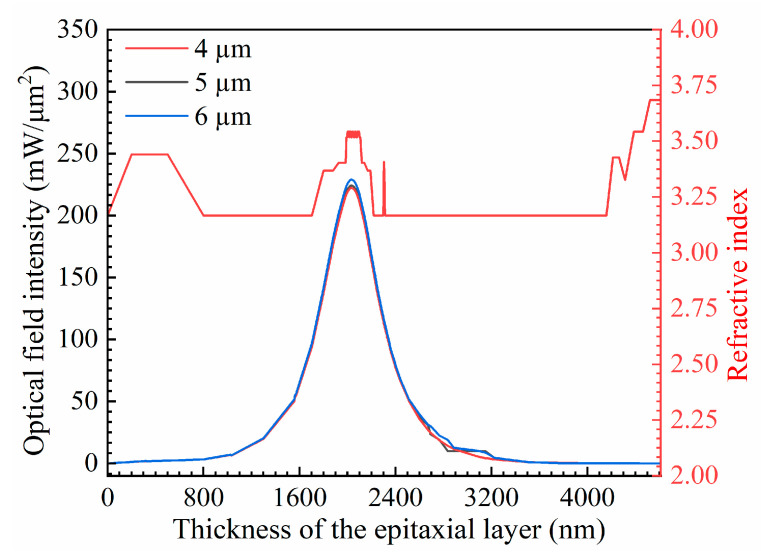
Optical field intensity and refractive index distributions at ridge widths of 4 μm, 5 μm, and 6 μm.

Figure 2 shows the optical field and refractive index distributions for various ridge widths. Notably, the optical field is confined to the active region. As the ridge width increases, the number of light beam reflections during transmission decreases, resulting in reduced energy loss and a gradual increase in the optical field intensity.

**Figure 3:**
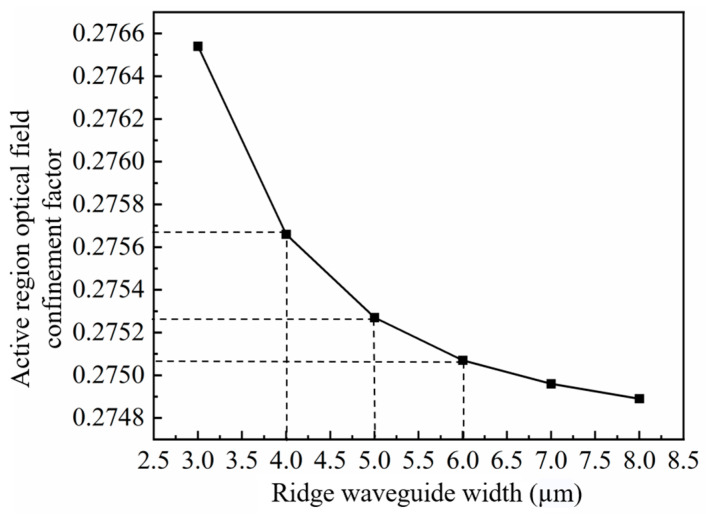
Relationship between active region optical confinement factor and ridge width.

The optical confinement factor (*Г*) is defined as the ratio of the light intensity in the active region to the light intensity produced by the entire device. A high optical confinement factor indicates strong optical confinement, which allows more photons to be confined in the active region, thereby enhancing the efficiency and stability of energy transfer [35]. A lower optical confinement factor can effectively decrease the heat generated in the quantum well region and reduce susceptibility to catastrophic optical mirror damage at a given power level, thereby enhancing the reliability of the device and allowing for higher output power. However, there exists a trade-off between achieving high gain and saturated output power. If the optical confinement factor is too small, the interaction between the optical signal and the active region may be insufficient to provide adequate gain, leading to a decrease in output power. Conversely, if the optical confinement factor is too large, it may lead to gain saturation and nonlinear effects, impacting both output power and signal quality. Thus, we investigated the effect of the ridge width (4 µm, 5 µm, and 6 µm) on the optical confinement factor. The refractive index of the ridge waveguide is correlated to the optical field intensity, as shown in Figure 2. Increasing the waveguide width reduces the optical confinement factor. According to the simulations, as the width of the ridge waveguide decreases, the optical confinement factor initially increases slightly from 0.27507 to 0.27527 and then increases to 0.27566 (Figure 3). Decreasing the ridge width results in more optical field energy being concentrated in the active region and thus increases the optical confinement factor.

**Figure 4:**
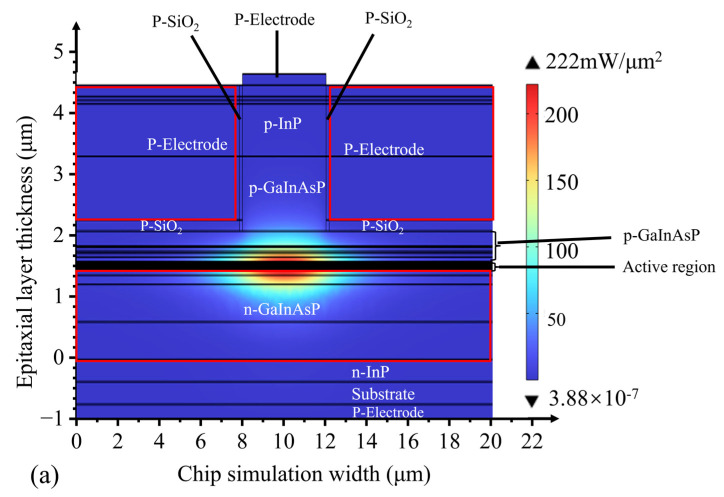

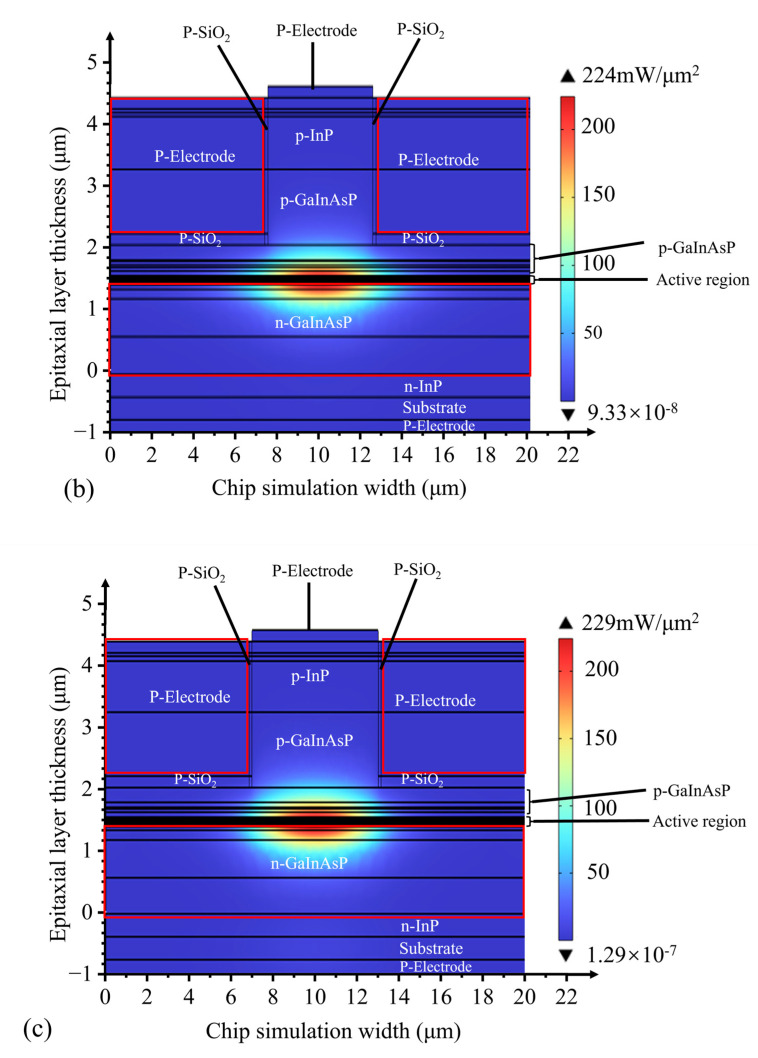
Optical field distributions for ridge widths of (**a**) 4 µm, (**b**) 5 µm, and (**c**) 6 µm.

To further analyze the light-field distribution in the waveguide structure, we employed finite element method (FEM) software to simulate the optical field within the active region. As shown in Figure 4, with the increase in ridge width, the optical field confinement factor of the active region decreases, leading to the broadening of the optical field mode towards the n-type waveguide layer. The distribution of the optical field mode significantly expands, which is beneficial for enhancing the saturated output power of the SOA device.

As SOAs are used as optical signal amplifiers, the ability of these components to amplify optical signals is an important index for evaluating SOA performance. The gain of the SOA increases with increasing injection current and concentration of injected carriers. At a given bias current, the output power of the SOA increases linearly with the input power; this linear relationship corresponds to the small-signal gain (*G*_0_), which is expressed as follows [36]:

(1) $$G_{0}=\exp[\left({Гg}_{0}-\alpha_{i}\right)L]$$

where $g_{0}$ is the material gain coefficient, $g_{0}$ is the internal loss coefficient, *τ* is the carrier lifetime, and *L* is the cavity length. In practice, a key parameter is the saturated output power ($P_{\mathrm{sat}}$), which is typically defined as the output power corresponding to a 3 dB decrease in gain, as expressed by [37]:

(2) $$P_{\mathrm{sat}}=\frac{\ln2}{1-2/G_{0}}\left(\frac{\mathrm{wd}}{Г}\right)\left(\frac{h\nu }{a\tau }\right)$$

**Figure 5:**
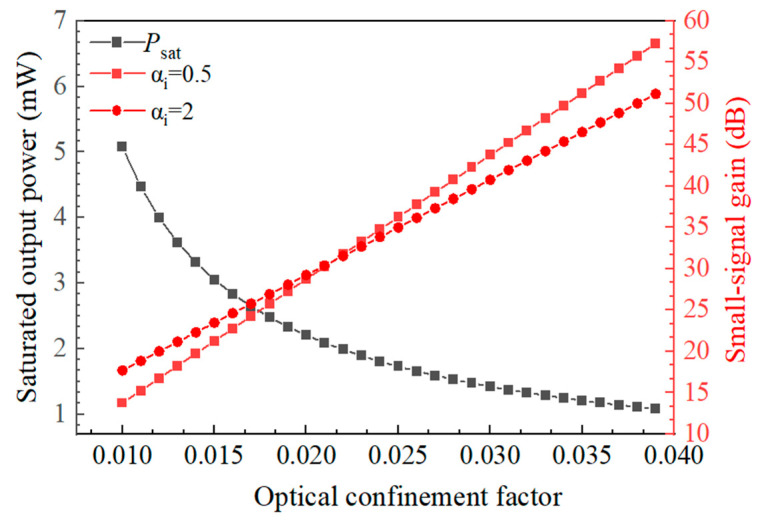
Saturated output power (*P*_sat_) and small-signal gain (*G*_0_) as functions of *Γ*.

The relationship between the saturated output power, the optical confinement factor, and the small-signal gain was simulated using Equation (2), where w is the ridge width of the device waveguide, d is the thickness of the active region, and a is the material differential gain. As shown in Figure 5, the saturated output power and small-signal gain exhibit opposite trends as the optical confinement factor increases. Therefore, SOA devices cannot simultaneously achieve a high saturated output power and a large small-signal gain.

**Figure 6:**
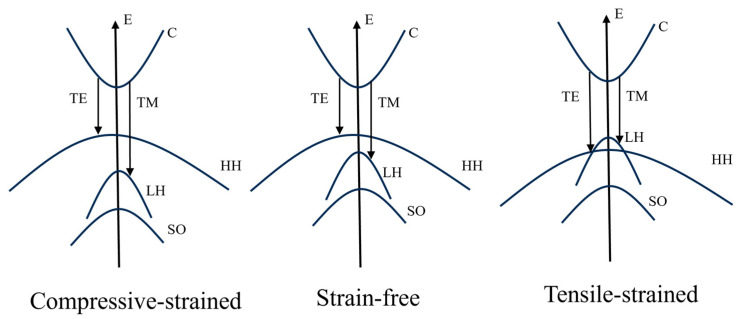
Energy band structures of strained quantum wells.

The quantum size effect increases the energy difference between the heavy-hole (HH) and light-hole (LH) bands at *k* = 0, causing the HH band to be positioned above the LH band. Consequently, electron transfer from the HH band to the conduction band is enhanced, whereas electron transfer from the LH band to the conduction band is suppressed. According to the Boltzmann equation, electron transfer from the HH band to the conduction band generates polarized photons in the TE mode. Thus, the material gain of the TE mode in the strain-free quantum well is greater than that of the TM mode (Figure 6). The introduction of tensor strain in the quantum well shifts the LH band to the top of the valence band and enhances electron jumping between the LH and conduction bands. This phenomenon increases the material gain of the TM mode.

To obtain polarization-insensitive SOAs, the optical gains of the two polarization modes are balanced by selecting an appropriate combination of strained quantum wells and potential barriers [38]. The optical gain can be accounted for using the following expression [39]:

(3) $$g\left(\hbar \omega \right)=C_{0}\sum_{n,m}{\left|I_{\mathrm{hm}}^{\mathrm{en}}\right|}^{2}\frac{2}{V}\sum_{\mathrm{kt}}{\left|\hat{e}\cdot p_{\mathrm{cv}}\right|}^{2}\delta \left(E_{\mathrm{hm}}^{\mathrm{en}}-E_{t}-\hbar \omega \right)\left(f_{c}^{n}-f_{v}^{m}\right)$$

(4) $$C_{0}=\frac{\pi e^{2}}{n_{r}c\varepsilon_{0}m_{0}^{2}\omega }$$

where ℏω is the energy of the incident photon; $\sum_{n,m}{\left|I_{hm}^{\mathrm{en}}\right|}^{2}$ is the wave function crossover integral; n and m refer to the conduction band sub-bands and valence band sub-bands, respectively; ${\left|\hat{e}\cdot p_{\mathrm{cv}}\right|}^{2}$ is a leptonic momentum matrix element; ${\left|\hat{e}\cdot p_{\mathrm{cv}}\right|}^{2}$ is the interband leptonic energy; and Et represents the partial energies of *k*_x_ and *k*_y_. Using the Fermi–Dirac function [39], the following can be obtained:

(5) $$f_{c}^{n}=\frac{1}{1+\exp\left[\left.{\left(E\right.}_{\mathrm{en}}+E_{g}+\frac{m_{r}^{*}}{m_{e}^{*}}E_{t}-F_{c}\right)/k_{B}T\right]}$$

(6) $$f_{v}^{m}=\frac{1}{1+\exp\left[\left.{\left(E\right.}_{\mathrm{hm}}-\frac{m_{r}^{*}}{m_{h}^{*}}E_{t}-F_{v}\right)/k_{B}T\right]}$$

(7) $$\frac{1}{m_{r}^{*}}=\frac{1}{m_{e}^{*}}+\frac{1}{m_{h}^{*}}$$

**Figure 7:**
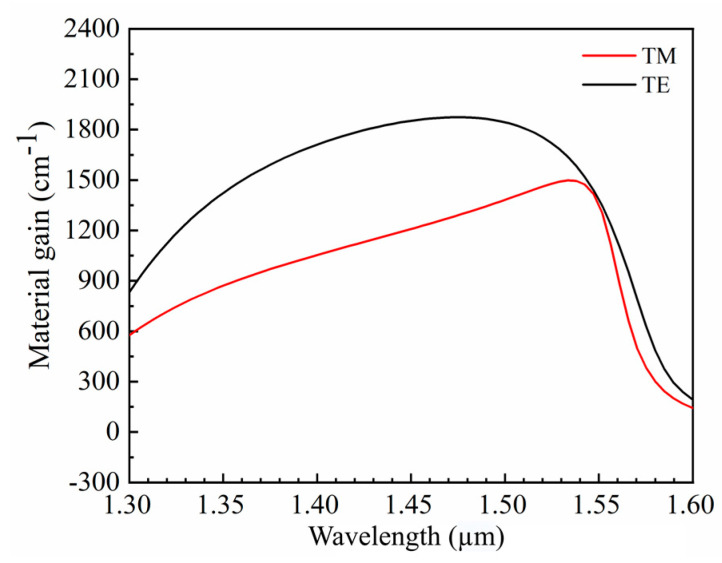
Material gain spectra.

The energies of the nth conduction band sub-band and the mth valence band sub-band are represented by $E_{\mathrm{en}}$ and $E_{\mathrm{hm}}$, respectively; $E_{g}$ is the band gap energy; $m_{r}^{*}$ is the effective mass approximation; $m_{e}^{*}$ and $m_{h}^{*}$ are the effective masses of an electron and a hole, respectively; $F_{c}$ and $F_{v}$ represent the quasi-Fermi energy levels of an electron and a hole, respectively; k_B_ is Boltzmann’s constant; and T is room temperature. The quantum barrier has a thickness of 12 nm, whereas the quantum well has a thickness of 10 nm. Figure 7 shows the material-gain simulation results for the TE and TM modes of five repeating quantum wells. These results indicate that the material gains of the TE and TM modes are nearly identical at 1550 nm, confirming the theoretical possibility of realizing polarization-insensitive SOAs. Additionally, the material gain of the TM mode exhibits a maximum value near 1550 nm. In contrast, the maximum material gain of the TE mode is greater and extends over a wider wavelength range, as determined by the material properties.

### 2.2. Device Preparation and Testing

**Figure 8:**
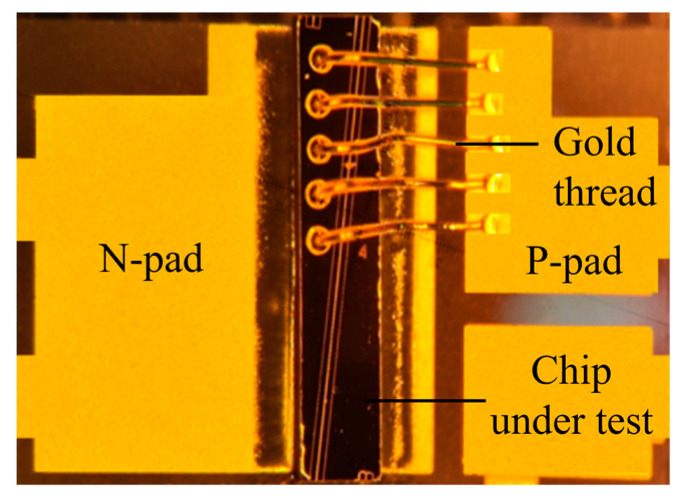
Image of the chip package.

The quality of an SOA device is closely related to the manufacturing process. To fabricate an SOA device, we first selected a substrate with a bright, flat, scratch-free surface. Epitaxial material growth was then performed via metal–organic chemical vapor deposition. Subsequent processes included photolithography, developing, etching, thinning, polishing, and coating. After plating with the transmittance-enhancing film, the residual reflectivity of the SOA was less than 0.005. Once growth was complete, the chip was dissociated into a bar and a single tube using high-precision scribing and lobbing machines. The single tube, with the n-side facing downwards, was then soldered to the heat sink to create an ohmic contact between the n-side and the heat sink. The p-side was connected to the heat sink via a gold wire to form another ohmic contact, as illustrated in Figure 8.

**Figure 9:**
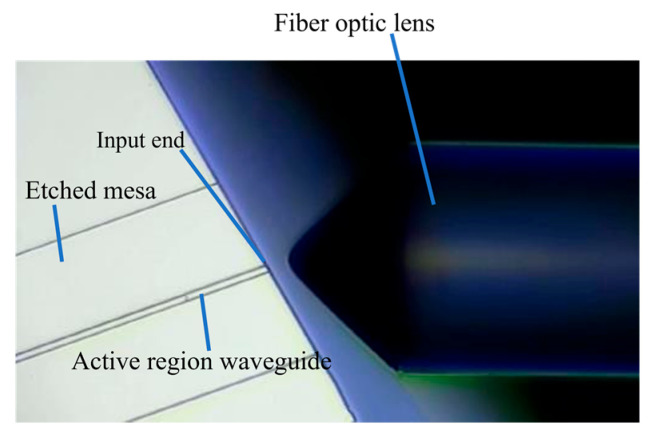
Experimental test setup.

**Figure 10:**
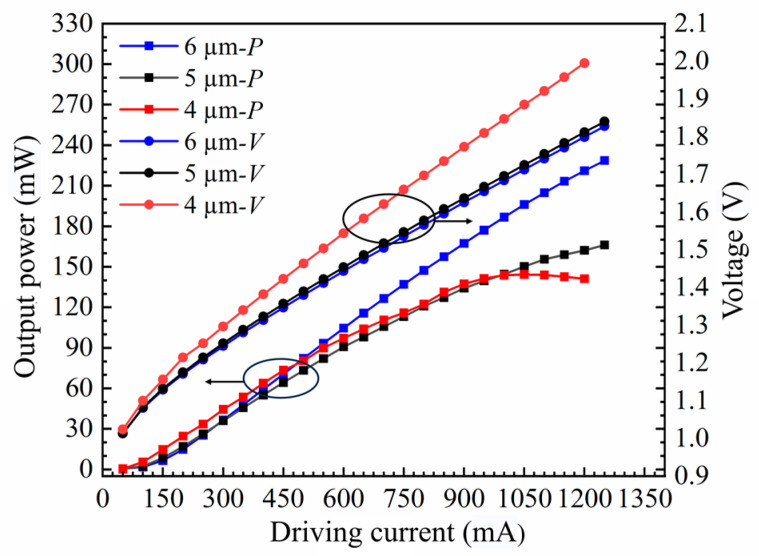
Power (*P*)–current (*I*)–voltage (*V*) plots for SOAs with various ridge widths.

The obtained chip was mounted on a pedestal and connected to a thermoelectric cooler to regulate the operating temperature precisely. A high-power, continuously tunable laser (EXFO T500S) was used as the seed source, and the laser light was fed into the SOA waveguide through a fiber optic lens. Owing to the 7° tile angle in the waveguide, the fiber optic lens must be incident to the waveguide end face at an angle of approximately 21° (Figure 9). For SOAs with different ridge widths, the dependence of the output power on the current was measured by varying the chip bias current (Figure 10). As the ridge width increases, the region where current injection occurs becomes larger. Consequently, the carrier concentration in the active region increases, ultimately resulting in a higher gain and output power. Slight differences occurred in the power range of 3 mW owing to minor variations in the coupling efficiency. However, upon increasing the current, the 4 μm device reached saturation quickly, with a maximum output power of 144.24 mW. The 5 μm device reached saturation at an output power of 166.16 mW, whereas no saturation was observed for the 6 μm device within the tested current range. This is in excellent agreement with the simulated predictions in Figure 3 and Figure 4. Additionally, the saturation power of the 6 μm device is 1.63 times higher than that of the 4 μm device.

**Figure 11:**
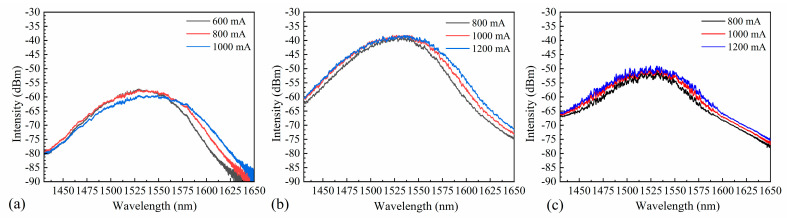
Spontaneous radiation spectra for SOAs with ridge widths of (**a**) 4 µm, (**b**) 5 µm, and (**c**) 6 µm.

Despite attempts to eliminate any remaining reflectivity, interference still affected the spontaneous radiation spectrum, as measured by coupling the light generated by the SOA to a spectrum analyzer without an external seed source. The spontaneous radiation spectra of chips with different ridge widths were obtained at three different currents (Figure 11). The bias currents for the SOAs with ridge widths of 5 µm and 6 µm were 0.8 A, 1 A, and 1.2 A. However, as the SOA with a ridge width of 4 µm could only withstand a maximum bias current of 1 A, bias currents of 0.6 A, 0.8 A, and 1 A were used. Increasing the bias current excites more carriers into the active region. As carriers with high energy levels produce spontaneous radiation, the intensity of spontaneous radiation increases. The residual reflectivity at the end face of the SOA causes some photons to return to the active region. This process excites carriers with high energy levels, inducing radiation and generating more photons, thereby enhancing the spontaneous radiation intensity.

#### 2.2.1. Saturated Output Power

**Figure 12:**
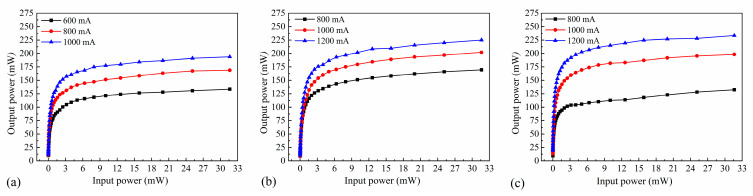
Output power curves at various input powers for SOAs with ridge widths of (**a**) 4 µm, (**b**) 5 µm, and (**c**) 6 µm.

After the addition of an external seed source, carriers with high energy levels are excited by photons, which induces the production of photons of the same wavelength as the seed source, resulting in signal-light amplification. The saturated output power and gain of SOAs with different ridge widths were measured by varying the input power and bias current of the signal light. As shown in Figure 12, the output power increases gradually with increasing bias current. For the SOA with a ridge width of 4 µm, the output power reaches 133.57 mW, 168.86 mW, and 193.76 mW at bias currents of 600 mA, 800 mA, and 1000 mA, respectively. For the SOA with a ridge width of 5 µm, the output power reaches 169.51 mW, 202.03 mW, and 224.96 mW at bias currents of 800 mA, 1000 mA, and 1200 mA, respectively. In the case of the SOA with a ridge width of 6 µm, the output power reaches 132.52 mW, 198.26 mW, and 233.41 mW at bias currents of 800 mA, 1000 mA, and 1200 mA, respectively. An increased bias current results in a corresponding increase in the carrier concentration within the SOA. Consequently, more carriers are located at higher energy levels and contribute to the excited radiation of the signal light, thereby enhancing the amplification ability and saturated output power of the SOA.

When the input power is sufficiently low, the output power increases gradually. The carriers in the gain region can support the number of carriers consumed by the excited radiation, resulting in the generation of more photons and a linear increase in the output power. When the input power is increased, more carriers in the SOA gain region are required to contribute to the excited radiation. However, the carriers in the gain region cannot be replenished over time, leading to slow growth of the output power and eventual saturation.

The maximum saturated output powers for ridge widths of 4 and 5 µm were 193.76 and 224.96 mW, respectively, whereas that for a ridge width of 6 µm was 233.41 mW—significantly higher. As the ridge width increases, more photons are propagated in the waveguide, thereby contributing to the excited radiation. However, a wider ridge weakens the interactions between the light wave and the waveguide, reducing loss of the light wave and increasing the saturated output power.

**Figure 13:**
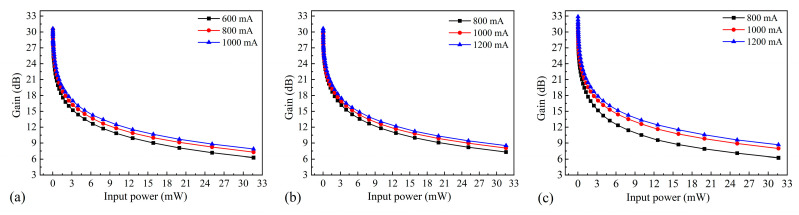
Dependence of gain on input power for SOAs with ridge widths of (**a**) 4 µm, (**b**) 5 µm, and (**c**) 6 µm.

The relationship between the gain and the input power is opposite to that observed for the output power (Figure 13). The input power of the seed source was gradually increased by keeping the TEC temperature constant, and the output power P_out_ (unit dBm) was measured. The peak gain of the device was obtained by the following formula: gain = P_out_ − P_in_. The maximum gain was obtained at an input power of 0.01 mW. Increasing the ridge width allows more photons to contribute to the excited radiation in the waveguide, resulting in stronger output light and higher gain. The maximum gains of the SOA with ridge widths of 4 µm, 5 µm, and 6 µm were 30.62 dB, 30.63 dB, and 32.89 dB, respectively. The SOA achieves higher gain at high currents because of the increase in the optical power of the signal, which requires more carriers for the excited radiation. Fewer carriers are supplied at low currents than at high currents. At a ridge width of 4 µm, the maximum gains were 29.99 dB, 30.52 dB, and 30.62 dB for bias currents of 600 mA, 800 mA, and 1000 mA, respectively. At a ridge width of 5 µm, the maximum gains were 29.41 dB, 29.65 dB, and 30.63 dB for bias currents of 800 mA, 1000 mA, and 1200 mA, respectively. At a ridge width of 6 µm, the maximum gains were 29.69 dB, 31.3 dB, and 32.89 dB for bias currents of 800 mA, 1000 mA, and 1200 mA, respectively. When the input power reaches a certain level, many carriers within the SOA are consumed, which decreases the number of carriers that can interact with the optical signal. Consequently, the gain gradually decreases, which causes a saturation effect that reduces the number of longitudinal beam modes and improves the optical transmission quality [40].

**Figure 14:**
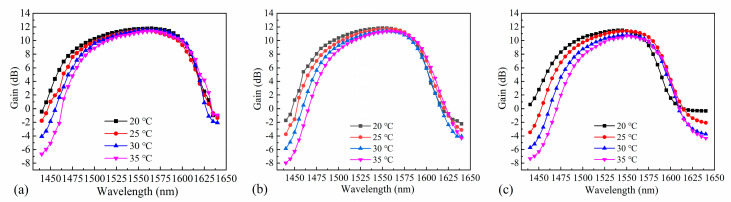
Effect of temperature on gain for SOAs with ridge widths of (**a**) 4 µm, (**b**) 5 µm, and (**c**) 6 µm.

**Figure 15:**
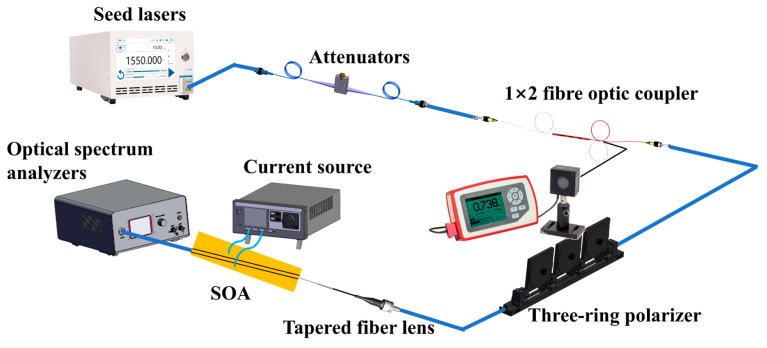
Schematic of experimental test setup.

Figure 14 shows the dependence of the gain on wavelength and temperature. Maintaining constant TEC temperature, set the input power to 10 dBm and adjust the drive current to the operational level. Then, vary the wavelength of the tuned laser from 1440 nm to 1640 nm with a step interval of 5 nm. Record the gain value at each wavelength and plot the gain curve corresponding to the wavelength change. Identify the wavelength range where the maximum recorded gain experiences a 3 dB drop; this range represents the 3 dB gain bandwidth (Figure 15). The center wavelength of the gain underwent a significant redshift upon increasing the operating temperature, which was caused by the nature of the material. Temperature changes can affect the refractive index of a material, leading to the emission of longer-wavelength photons. Light absorption and amplification by semiconductors are enhanced at low temperatures owing to the electronic structures and optical properties of these materials. Additionally, low temperatures promote carrier binding in quantum wells, resulting in higher gains. As the device temperature increases, the energy of the carriers also increases, which decreases the binding force of the quantum well. Consequently, carriers can escape more easily from the quantum well, which reduces the gain. Moreover, high temperatures intensify lattice vibrations, resulting in stronger carrier–phonon interactions and thus fewer carrier–photon interactions, which further reduces the gain. Furthermore, increased lattice vibrations accelerate the carrier complexation rate, resulting in a shorter carrier lifetime within the quantum well. This behavior results in a shorter time for carrier–photon interactions and ultimately decreases the gain. The device with a ridge width of 4 µm exhibited maximum gains of 11.79 dB, 11.47 dB, 11.54 dB, and 11.35 dB at 20 °C, 25 °C, 30 °C, and 35 °C, respectively. The device with a ridge width of 5 µm exhibited maximum gains of 11.85 dB, 11.7 dB, 11.39 dB, and 11.35 dB at 20 °C, 25 °C, 30 °C, and 35 °C, respectively. The device with a ridge width of 6 µm exhibited maximum gains of 11.5 dB, 11.372 dB, 10.854 dB, and 10.695 dB at 20 °C, 25 °C, 30 °C, and 35 °C, respectively. At higher temperatures, carriers move more vigorously and have a wider energy distribution. Thus, fewer carriers contribute to the excited radiation in the presence of the signal light, which narrows the width of the gain spectrum. Furthermore, the operational temperature affects the dynamic characteristics of the quantum-well SOAs.

#### 2.2.2. Small-Signal Gain

**Figure 16:**
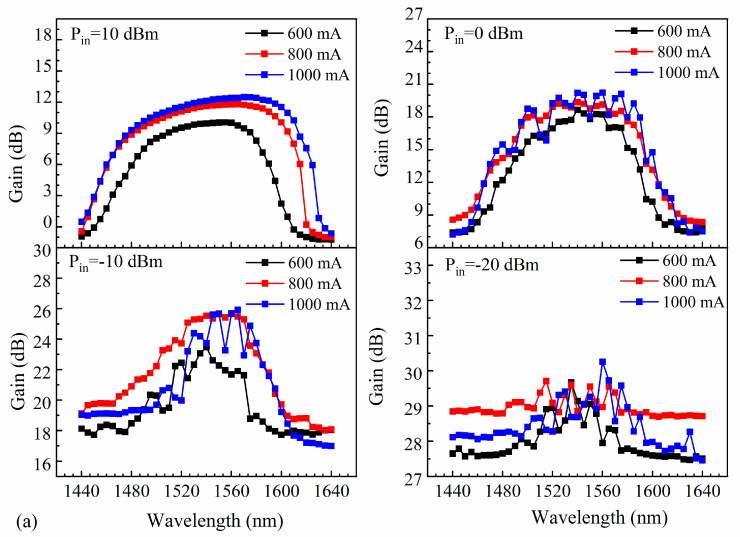

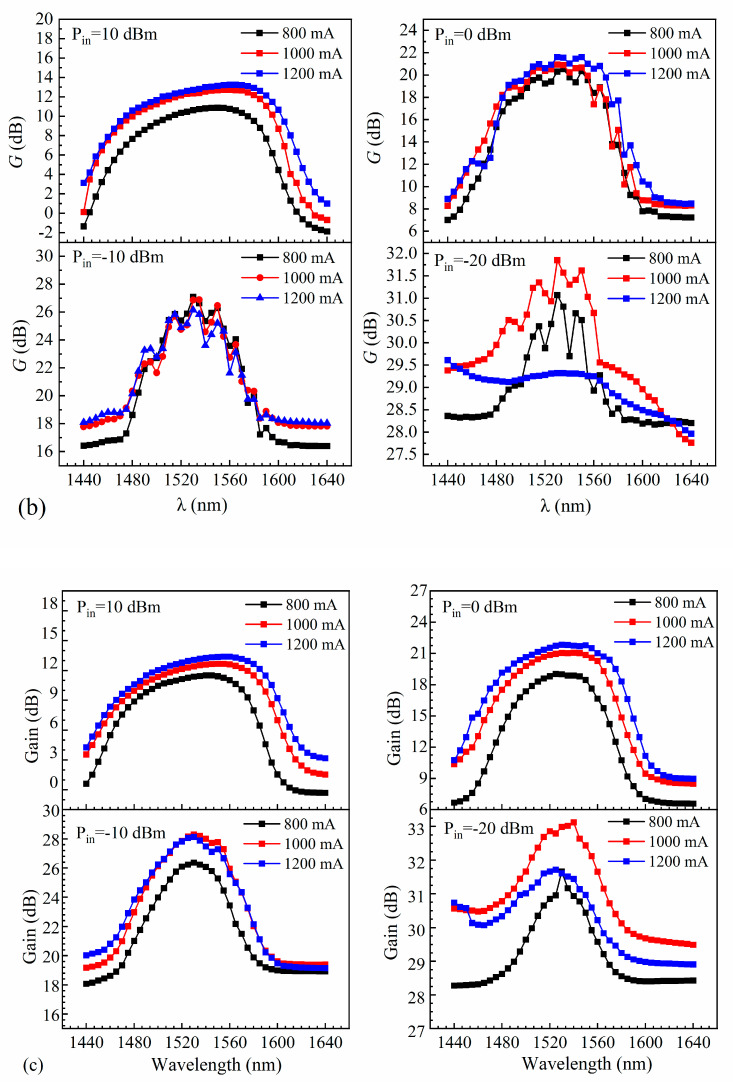
Gain at various input powers for SOAs with ridge widths of (**a**) 4 µm, (**b**) 5 µm, and (**c**) 6 µm.

Figure 16 shows the gain spectra obtained at various bias currents and input powers. Maintaining constant TEC temperature, adjust the input power to the appropriate values (Pin = 10 dBm, 0 dBm, −10 dBm, and −20 dBm), and set the drive current to the working levels (600 mA, 800 mA, and 1000 mA for a ridge width of 4 µm, and 800 mA, 1000 mA, and 1200 mA for ridge widths of 5 µm and 6 µm). Then, vary the wavelength of the tuned laser from 1440 nm to 1640 nm with a step interval of 5 nm. Record the gain value at each wavelength and plot the gain curve corresponding to the wavelength change. A flatter gain spectrum was obtained over a wavelength range of 60 nm at an input power of 10 dBm and a bias current of 600 mA (Figure 16a). Similarly, at bias currents of 800 mA and 1000 mA, the gain spectra were flatter over wavelength ranges of 85 nm and 100 nm, respectively. For ridge widths of 5 µm and 6 µm, the wavelength range over which the gain is flat becomes wider at a higher bias current (Figure 16b,c). A larger bias current provides more carriers that can participate in excited emission, resulting in the gain being more uniform over a wider range of wavelengths.

The effect of varying the input power (10 dBm, 0 dBm, −10 dBm, and −20 dBm) was also investigated. At an input power of 10 dBm, the SOAs with ridge widths of 4 µm, 5 µm, and 6 µm exhibited flatter gain spectra. However, as the input power decreased, the gains of the SOAs increased and exhibited greater fluctuations. The gain of an SOA is closely related to the carrier concentration. At low input powers, the carrier concentration within an SOA is more susceptible to external factors, which can induce fluctuations in the carrier concentration and, consequently, the gain. The CH effect (carrier heating effect) of an SOA demonstrates the interaction between photons and carriers at the edges of the energy bands in the presence of a signal light. However, the temperature of the carriers at the edge of the energy band is lower than the average temperature. Consequently, the remaining carriers have a higher temperature, which affects the gain characteristics of the SOA. This effect becomes more significant at low input powers. The nonlinear effects of the SOA may also lead to instability. The nonlinear effects may vary depending on the wavelength of the optical signal. Therefore, changing the wavelength of the seed source may also influence the nonlinear effects, leading to increased gain fluctuations. As the ridge width of the SOAs increases, the heat generated by current injection may be more evenly distributed throughout the SOA structures, which can reduce the impact of thermal effects on the gain fluctuations.

At high input powers, increasing the bias current causes more carriers in the SOA gain region to contribute to stimulated emission and emit photons. Therefore, when the power of the input signal is constant, the gain of the SOA increases with increasing bias currents. As the input power decreases, the number of incident photons also decreases. Even if the bias current and number of carriers are increased, the amount of photons available to interact with the carriers is insufficient, resulting in no further increase in gain. Additionally, the CH effect causes an increase in the carrier temperature, leading to decreases in the complexation rate and carrier mobility, which ultimately reduces the SOA gain.

**Figure 17:**
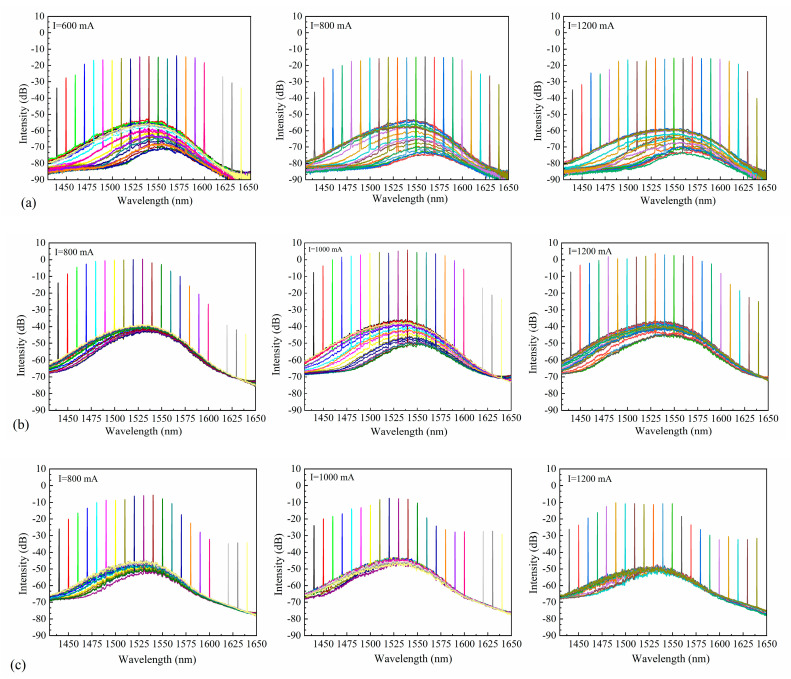
Output power spectra at different currents for SOAs with ridge widths of (**a**) 4 µm, (**b**) 5 µm, and (**c**) 6 µm.

Using the same methodology as described earlier, the TEC temperature was held constant while adjusting both the bias current of the SOA and the output wavelength of the seed source within the wavelength tuning range of 1440 nm to 1640 nm. The stimulated radiation spectrum of the SOA was then measured, as illustrated in Figure 17. The different coloured curves represent spectral curves at different wavelengths. It was observed that a smaller ridge width in the SOA led to a stronger confinement effect on the optical field. Consequently, an SOA with a ridge width of 4 µm exhibited a flatter gain spectrum over a wider wavelength range. As the ridge width increased, the spectral flatness weakened, yet the gain flatness of the SOA under various bias currents showed no significant alteration. 

Linewidth is a relatively important metric for SOAs. The spontaneous radiation phenomenon of SOAs results in the generation of mutually independent photons, which causes linewidth broadening. Coherent light generated by excited radiation can attenuate the propagation process, which also causes linewidth broadening. These issues can be effectively mitigated by optimizing various conditions, such as the SOA operating temperature and pump power.

**Figure 18:**
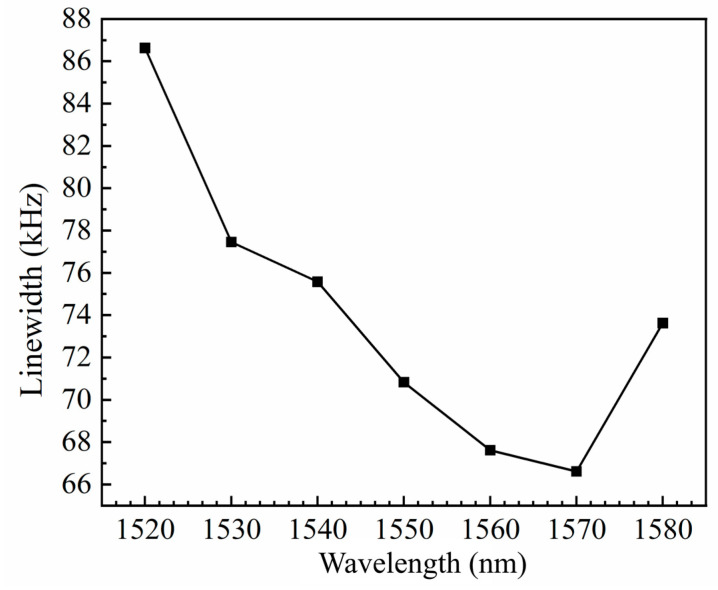
The variation in seed source linewidth with wavelength.

First, the linewidth of the seed-source laser was investigated. For these measurements, the power of the seed source entering the linewidth test system was reduced to less than 2 mW, the resolution bandwidth of the spectrometer was 300 Hz, and the sweep width was 1 MHz. Twenty scans were collected, and the average was calculated to obtain linewidth maps of the seed source at different wavelengths. A Lorentzian fit was applied to the measured linewidth maps to determine the linewidth values, as shown in Figure 18.

**Figure 19:**
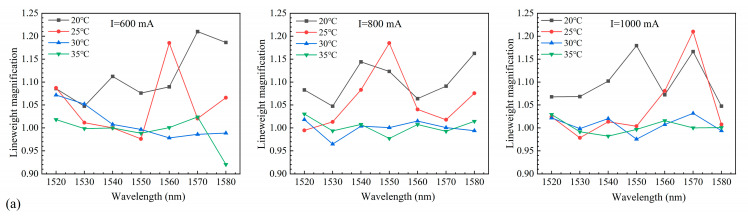

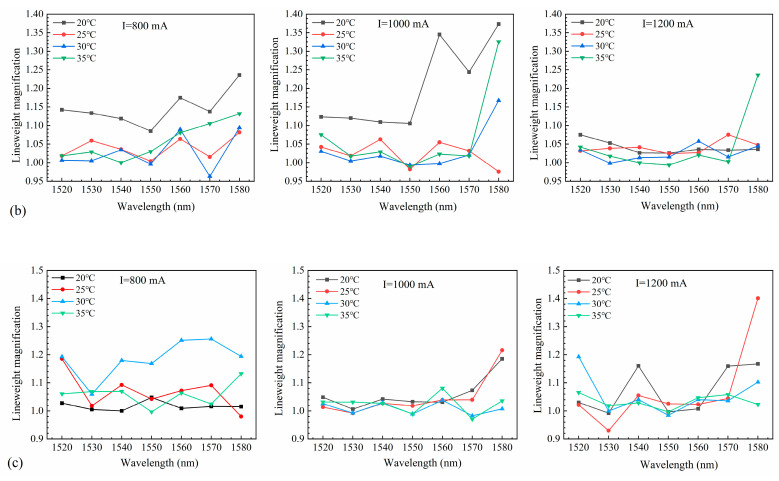
Linewidth magnification at different temperatures and input currents for SOAs with ridge widths of (**a**) 4 µm, (**b**) 5 µm, and (**c**) 6 µm.

After the seed source was amplified by the SOAs, the power was attenuated to less than 2 mW to access the linewidth test system, and measurements were performed using the above-described settings. The linewidths at different operating temperatures and currents were obtained by Lorentzian fitting, and the linewidth magnification is shown in Figure 19. Although the chip was affected by external factors and the linewidths fluctuated considerably, significant linewidth broadening did not occur. Notably, the linewidths after SOA amplification were less than 1.4 times those of the seed-source laser.

The noise figure is an important measure of the noise performance of an SOA. This parameter, which indicates the degradation degree of the signal-to-noise ratio caused by the signal passing through the SOA, is defined in [41] as follows:

(8) $$\mathrm{NF}=\frac{(S/N{)}_{\mathrm{in}}}{(S/N{)}_{\mathrm{out}}}$$

where $(S/N{)}_{\mathrm{in}}$ and $(S/N{)}_{\mathrm{out}}$ are the signal-to-noise ratios of the SOA input and output, respectively. For the SOA, the noise mainly consisted of scattered grain and beat noise. Scattered grain noise is a random wave of energy caused by the collision of charge carriers and lattices or the thermal vibration of charge carriers and photons at a given temperature, which is unavoidable. Beat noise can be divided into beat noise between spontaneously emitted photons and beat noise between the signal light and spontaneously emitted photons. To reduce the noise and improve the quality of the output light, the residual reflectivity of the end face of the SOA should be minimized.

**Figure 20:**
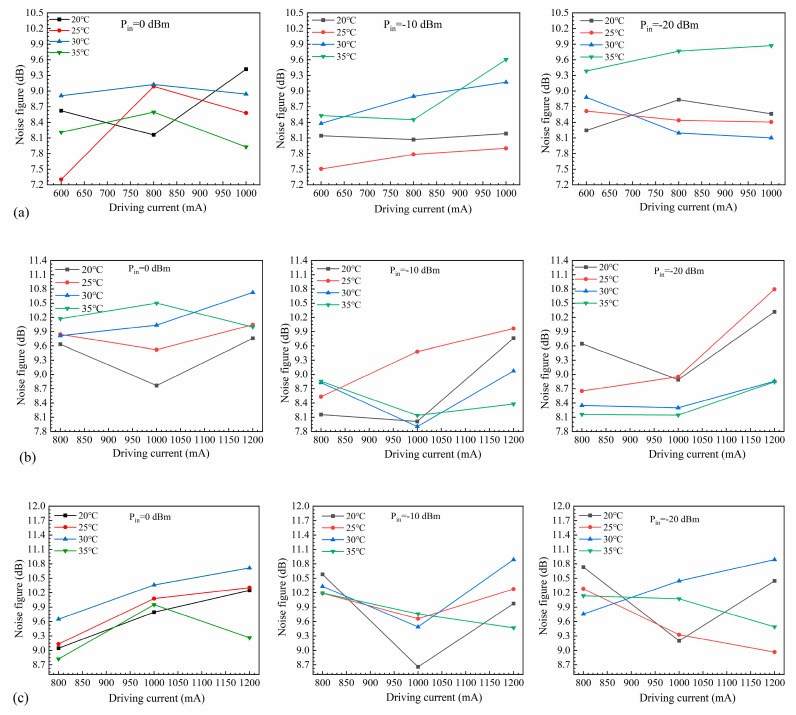
Noise figure at different temperatures for SOAs with ridge widths of (**a**) 4 µm, (**b**) 5 µm, and (**c**) 6 µm.

Noise figure measurements were performed using a spectral analyzer, where the signal light and the light amplified by the SOA were fed into the spectral analyzer for EDFA-NF analysis. The input/output power was compensated according to the analysis results, and the final results were obtained by performing the EDFA-NF analysis again. We measured the noise figure of the SOA device under different operating conditions by varying the operating temperature, the bias current, and the input power (Figure 20). As the chip was not encapsulated and thus affected by external factors, the noise figure was unstable and sometimes high.

**Figure 21:**
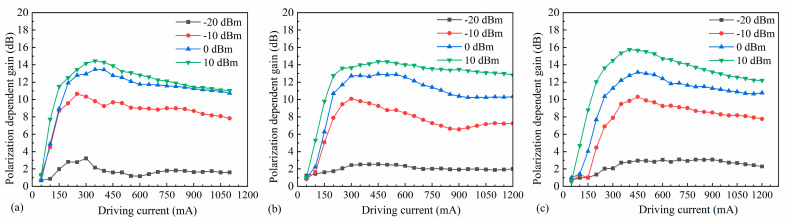
Gain polarizability at different input powers for SOAs with ridge widths of (**a**) 4 µm, (**b**) 5 µm, and (**c**) 6 µm.

The TEC temperature was held constant while varying the SOA bias current and the power of the seed source. The maximum output power of the TE polarization state (in dBm form) and the minimum output power of the TM polarization state (also in dBm form) were measured by adjusting the polarization controller. The TE polarization gain (Gain _pol-TE_) and the TM polarization gain (Gain _pol-TM_) were obtained. The polarization-dependent gain (PDG) of the device could then be obtained using the following formula: PDG = Gain _pol-TE_ − Gain _pol-TM_. Polarization insensitivity is a fundamental requirement for SOAs in optical amplification, signal processing, and optical switching [42]. Hence, the polarization characteristics of the 4 µm, 5 µm, and 6 µm SOA devices were systematically tested in this study, as shown in Figure 21. With the increase in driving current, the PDG exhibits an overall increasing trend followed by a gradual decrease. Under the same driving current, the PDG increases with increasing input power. At a small signal input of −20 dBm, the overall PDG remains below 3 dB, indicating low polarization sensitivity. Moreover, with increasing ridge width, the PDG shows a slow increase. For instance, at a −20 dBm input, the lowest PDG for the 4 µm width is only 1.21 dB, as shown in Figure 21a. However, when the input power reaches 10 dBm, all three ridge widths exhibit significantly higher PDGs, with the 6 µm width reaching a maximum of 15.74 dB. Combining the simulations conducted earlier, it is evident that strain adjustment in the LH band effectively reduces the mode gain difference between TE and TM modes. Nonetheless, increased strain significantly raises the difficulty of epitaxial growth. Thus, as seen in Figure 7, the TE mode still exhibits slightly higher gain in the design proposed in this study. Furthermore, testing also reveals that the PDG is not only related to the epitaxial structure but also to the active waveguide morphology. As the ridge width increases, the optical field mode changes, with the mode gain of the TE mode increasing faster than that of the TM mode. Therefore, based on the epitaxial structure design discussed above, small signal inputs and narrow ridge waveguide structures can effectively reduce the PDG of the SOA.

The developed SOA achieved polarization insensitivity while providing a high saturation output power and gain. The performance characteristics of various previously reported SOAs are summarized in Table 1. In 1994, NTT Opto-electronics Laboratories in Japan reported the use of tension–strain quantum wells as polarimetric insensitive SOAs in the active region, with a saturated output power of 14 dBm, a gain of 27.5 dB, and a polarization sensitivity of less than 0.5 dB at a 1560 nm wavelength [43]. In 2011, Japan’s Fujitsu used a cylindrical quantum dot structure to achieve a saturated output power of 18.5 dBm and a gain of 8 dB, and its polarization can reach a minimum of 0.4 dB [44]. In 2014, the Centre for Research in Photonics proposed an asymmetric MQW SOA operating at the 1.35 μm wavelength band. When the input power was −20 dBm, the PDG was less than 0.5 dB, with a peak gain of 20 dB. The saturated output power for the TE mode and the TM mode were 18 dBm and 22 dBm, respectively, with a 3 dB gain bandwidth of 100 nm [31]. In 2016, Huazhong University of Science and Technology proposed an integrated InGaAsP-InP/SOI polarization-insensitive SOA operating at the 1.55 μm wavelength band. When the input power was −20 dBm, the polarization-dependent gain (PDG) was less than 0.5 dB, but the gain was only 10.6 dB, with an output power tested to be −0.6 dBm, and a 3 dB gain bandwidth of 60 nm [45]. In 2018, the Technion-Israel Institute of Technology reported a high-gain quantum dot SOA that realized maximum gains of 22 dB at 50 °C and 15 dB at 100 °C, with a saturation output power of 9.62 dBm [46]. In 2020, Eindhoven University of Technology reported an MQW SOA with maximum gains of 7 dB and 16 dB at 1500 nm for bias currents of 50 mA and 80 mA, respectively, and a polarization sensitivity of less than 3 dB. At 1540 nm, for bias currents of 50 and 80 mA, the maximum gains were 12 dB and 20 dB, respectively, and the polarization sensitivity was less than 5 dB [19]. In 2020, Kobe University in Japan proposed a polarization-insensitive SOA operating at the 1.1 μm wavelength band, using closely stacked InAs/GaAs quantum dots. When the input power was -33 dBm, the PDG was less than 1 dB, with a maximum gain of approximately 30 dB, an output power of −3 dBm, and a 3 dB gain bandwidth exceeding 100 nm [47]. In 2021, Eindhoven University of Technology in the Netherlands proposed a polarization-insensitive bulk SOA operating at the 1.31 μm wavelength band for active–passive photonic circuits. When the input power was −20 dBm, the PDG was less than 1.5 dB, with an average gain greater than 20 dB, a saturated output power of 11 dBm, and a 3 dB gain bandwidth of approximately 40 nm [48].

Although previous research reports have shown that achieving a low PDG with small signal inputs is possible, it has been challenging to achieve gains exceeding 30 dB and saturated output powers beyond 18.5 dBm, with a 3 dB gain bandwidth surpassing 100 nm. However, in this study, while ensuring a device with a 3 dB gain bandwidth of 140 nm, a maximum gain of 32.89 dB, and a saturated output power of 23.38 dBm, we have still managed to maintain a PDG below 3 dB. This lays a solid foundation for ultra-high-speed, wideband coherent optical communication applications.

## 3. Conclusions

In summary, this paper proposed a novel SOA device tailored to the requirements of next-generation ultra-high-speed coherent optical communications, characterized by high power, low polarization sensitivity, and a wide gain spectrum. Through meticulous optimization of the strain characteristics within the active region to minimize material mode gain discrepancies and enhance gain bandwidth, coupled with the optimization of the relationship between the optical confinement factor of the active region and the ridge width to achieve an asymmetric large cavity, the device’s saturated output power was significantly elevated. A maximum saturated output power of 233 mW was achieved with a ridge width of 6 µm. When the ridge width was 4 µm, the maximum gain was 32.89 dB at an input power of −20 dBm, with the gain being flattest within the wavelength range of 1475 nm to 1615 nm. Overall, this study provided new insights and approaches for the further development of core optical amplifiers in the field of optical communications, demonstrating significant practical and scientific significance.

## Figures and Tables

**Table 1:** Performance comparison with previously reported SOAs.

Publication Year	Research Unit	Gain	Saturation Output Power	Polarization-Dependent Gain	Gain Bandwidth
1994 [43]	NTT Opto-electronics Laboratories	27.5 dB	14 dBm	<0.5 dB@13.5 dBm	/
2011 [44]	Fujitsu Laboratories Ltd.	8 dB	18.5 dBm	0.4 dB@10 dBm	~30 nm
2014 [31]	Photonics Technology Laboratory, Centre for Research in Photonics	20 dB	22 dBm	<0.5 dB@-20 dBm	100 nm
2016 [45]	Huazhong University of Science and Technology	10.6 dB	Only −0.6 dBm was tested	<0.5 dB@-20 dBm	60 nm
2018 [46]	Technion-Israel Institute of Technology	22 dB	9.62 dBm	/	~30 nm
2020 [19]	Eindhoven University of Technology	20 dB	7.4 dBm	<3 dB	~35 nm
2020 [47]	Kobe University	~30 dB	~3 dBm	<1 dB@-33 dBm	>100 nm
2021 [48]	Eindhoven University of Technology	20 dB	11 dBm	1.5 dB@-20 dBm	~40 nm
**202** **4**	**This work**	**32.89 dB**	**23.38 dBm**	<**3 dB**@-20 dBm	>**140 nm**

## Data Availability

The data presented in this study can be found in the article.

## References

[1] Moges T.H., Lakew D.S., Nguyen N.P., Dao N.-N., Cho S. (2023). Cellular Internet of Things: Use cases, technologies, and future work. Internet Things.

[2] Pham Q.-V., Fang F., Ha V.N., Piran M.J., Le M., Le L.B., Hwang W.-J., Ding Z. (2020). A survey of multi-access edge computing in 5G and beyond: Fundamentals, technology integration, and state-of-the-art. IEEE Access.

[3] Ji Y., Zhang J., Xiao Y., Liu Z. (2019). 5G flexible optical transport networks with large-capacity, low-latency and high-efficiency. China Commun..

[4] Wang Z., Du Y., Wei K., Han K., Xu X., Wei G., Tong W., Zhu P., Ma J., Wang J. (2022). Vision, application scenarios, and key technology trends for 6G mobile communications. Sci. China Inf. Sci..

[5] Petkova R., Bozhilov I., Manolova A., Tonchev K., Poulkov V. (2024). On the Way to Holographic-Type Communications: Perspectives and Enabling Technologies. IEEE Access.

[6] Yu J., Wu Y. (2022). High-speed optical fiber communication in China. ACS Photonics.

[7] Kikuchi K. (2015). Fundamentals of coherent optical fiber communications. J. Light. Technol..

[8] Winzer P.J., Neilson D.T., Chraplyvy A.R. (2018). Fiber-optic transmission and networking: The previous 20 and the next 20 years. Opt. Express.

[9] Sobhanan A., Anthur A., O’Duill S., Pelusi M., Namiki S., Barry L., Venkitesh D., Agrawal G.P. (2022). Semiconductor optical amplifiers: Recent advances and applications. Adv. Opt. Photonics.

[10] Pham C., Duport F., Brenot R., Paret J.-F., Garreau A., Gomez C., Fortin C., Mekhazni K., van Dijk F. (2020). Modulation of a high power semiconductor optical amplifier for free space communications. J. Light. Technol..

[11] El-Hageen H.M., Alatwi A.M., Zaki Rashed A.N. (2024). High-speed signal processing and wide band optical semiconductor amplifier in the optical communication systems. J. Opt. Commun..

[12] Renaudier J., Napoli A., Ionescu M., Calo C., Fiol G., Mikhailov V., Forysiak W., Fontaine N., Poletti F., Poggiolini P. (2022). Devices and fibers for ultrawideband optical communications. Proc. IEEE.

[13] Wang B. A research on all-optical wavelength conversion technology based on SOA. Proceedings of the 2021 11th International Conference on Power, Energy and Electrical Engineering (CPEEE).

[14] Rasoulzadehzali A., Kleijn S., Augustin L.M., Stabile R., Calabretta N. Low Polarization Sensitive Semiconductor Optical Amplifier Co-Integrated with Passive Waveguides for Optical Datacom and Telecom Networks. Proceedings of the 22nd European Conference on Integrated Optics (ECIO 2020).

[15] Ma W., Tan S., Wang K., Guo W., Liu Y., Liao L., Zhou L., Zhou J., Li X., Liang L. (2020). Practical two-dimensional beam steering system using an integrated tunable laser and an optical phased array. Appl. Opt..

[16] Blumenthal D.J. Optical packet switching. Proceedings of the 17th Annual Meeting of the IEEELasers and Electro-Optics Society, LEOS 2004.

[17] Ó Dúill S.P., Landais P., Barry L.P. (2017). Estimation of the performance improvement of pre-amplified PAM4 systems when using multi-section semiconductor optical amplifiers. Appl. Sci..

[18] Zali A.R., Kleijn S., Augustin L., Tessema N.M., Prifti K., Stabile R., Calabretta N. (2022). Design and fabrication of low polarization dependent bulk SOA co-integrated with passive waveguides for optical network systems. J. Light. Technol..

[19] Zali A.R., Stabile R., Calabretta N. Low polarization dependent MQW semiconductor optical amplifier with tensile-strained-barrier design for optical datacom and telecom networks. Proceedings of the 2020 22nd International Conference on Transparent Optical Networks (ICTON).

[20] Forsyth D.I., Mahad F.D. (2020). Semiconductor optical amplifiers: Present and future applications. Recent Developments in Optical Communication and Networking.

[21] Keyvaninia S., Beckerwerth T., Zhou G., Gruner M., Ganzer F., Ebert W., Mutschall S., Seeger A., Runge P., Schell M. (2019). Novel photodetector chip for polarization diverse detection. J. Light. Technol..

[22] Ostadrahimi M., Zakaria A., LoVetri J., Shafai L. (2013). A near-field dual polarized (TE–TM) microwave imaging system. IEEE Trans. Microw. Theory Tech..

[23] Walker J.D., Dijaili S.P., Ratowsky R.P. (2003). Polarization Insensitive Semiconductor Optical Amplifier. U.S. Patent.

[24] Aalto T., Solehmainen K., Harjanne M., Kapulainen M., Heimala P. (2006). Low-loss converters between optical silicon waveguides of different sizes and types. IEEE Photonics Technol. Lett..

[25] Knights A.P., Jessop P.E. (2018). Silicon waveguides for integrated optics. Optical Waveguides.

[26] Morito K., Ekawa M., Watanabe T., Kotaki Y. (2003). High-output-power polarization-insensitive semiconductor optical amplifier. J. Light. Technol..

[27] Michie C., Kelly A., McGeough J., Armstrong I., Andonovic I., Tombling C. (2006). Polarization-insensitive SOAs using strained bulk active regions. J. Light. Technol..

[28] Nkanta J.E., Maldonado-Basilio R., Khan K., Benhsaien A., Abdul-Majid S., Zhang J., Hall T.J. (2013). Low polarization-sensitive asymmetric multi-quantum well semiconductor amplifier for next-generation optical access networks. Opt. Lett..

[29] Juodawlkis P.W., Plant J.J., Loh W., Missaggia L.J., O’Donnell F.J., Oakley D.C., Napoleone A., Klamkin J., Gopinath J.T., Ripin D.J. (2011). High-power, low-noise 1.5-μm slab-coupled optical waveguide (SCOW) emitters: Physics, devices, and applications. IEEE J. Sel. Top. Quantum Electron..

[30] Lysak V., Kawaguchi H., Sukhoivanov I. (2005). Gain spectra and saturation power of asymmetrical multiple quantum well semiconductor optical amplifiers. IEE Proc.-Optoelectron..

[31] Nkanta J.E., Maldonado-Basilio R., Abdul-Majid S., Zhang J., Hall T.J. Asymmetric MQW semiconductor optical amplifier with low-polarization sensitivity of over 90-nm bandwidth. Proceedings of the Broadband Access Communication Technologies VIII.

[32] Cheng Q., Rumley S., Bahadori M., Bergman K. (2018). Photonic switching in high performance datacenters. Opt. Express.

[33] Besancon C., Néel D., Make D., Ramírez J.M., Cerulo G., Vaissiere N., Bitauld D., Pommereau F., Fournel F., Dupré C. (2021). AlGaInAs multi-quantum well lasers on silicon-on-insulator photonic integrated circuits based on InP-seed-bonding and epitaxial regrowth. Appl. Sci..

[34] Zhukov A., Kryzhanovskaya N., Moiseev E., Dragunova A., Nadtochiy A., Maximov M., Gordeev N.Y. (2022). Increase in the Efficiency of a Tandem Semiconductor Laser–Optical Amplifier Based on Self-Organizing Quantum Dots. Semiconductors.

[35] Tang H., Yang C., Qin L., Liang L., Lei Y., Jia P., Chen Y., Wang Y., Song Y., Qiu C. (2023). A Review of High-Power Semiconductor Optical Amplifiers in the 1550 nm Band. Sensors.

[36] Agarwal V., Agrawal M. Characterization and optimization of semiconductor optical amplifier for ultra high speed applications: A review. Proceedings of the 2018 Conference on Signal Processing And Communication Engineering Systems (SPACES).

[37] Yu S., Gallet A., Elfaiki H., Dahdah N.E., Brenot R. Novel semiconductor optical amplifier with large gain and high saturation output power. Proceedings of the 2021 European Conference on Optical Communication (ECOC).

[38] Carrère H., Truong V., Marie X., Brenot R., De Valicourt G., Lelarge F., Amand T. (2010). Large optical bandwidth and polarization insensitive semiconductor optical amplifiers using strained InGaAsP quantum wells. Appl. Phys. Lett..

[39] Chuang S.L. (2012). Physics of Photonic Devices.

[40] Li X., Liang L., Wang L.J., Qin L., Chen Y.Y., Wang Y.B., Song Y., Lei Y.X., Jia P., Zeng Y.G. (2021). Monolithic integrated semiconductor optical amplifier with broad spectrum, high power, and small linewidth expansion. IEEE Access.

[41] Said Y., Rezig H., Bouallegue A. (2008). Analysis of noise effects in long semiconductor optical amplifiers. Open Opt. J..

[42] Huang L., Huang D., Chen J., Liu D., Zhang X. (2006). Analysis of a semiconductor optical amplifier with polarization-insensitive gain and polarization-insensitive phase modulation. Semicond. Sci. Technol..

[43] Magari K., Okamoto M., Suzuki Y., Sato K., Noguchi Y., Mikami O. (1994). Polarization-insensitive optical amplifier with tensile-strained-barrier MQW structure. IEEE J. Quantum Electron..

[44] Yasuoka N., Ebe H., Kawaguchi K., Ekawa M., Sekiguchi S., Morito K., Wada O., Sugawara M., Arakawa Y. (2011). Polarization-insensitive quantum dot semiconductor optical amplifiers using strain-controlled columnar quantum dots. J. Light. Technol..

[45] Zhu Z., Li X., Xi Y. (2016). A polarization insensitive semiconductor optical amplifier. IEEE Photonics Technol. Lett..

[46] Eyal O., Willinger A., Mikhelashvili V., Banyoudeh S., Schnabel F., Sichkovsky V., Reithmaier J.P., Eisenstein G. High Performance 1550 nm Quantum Dot Semiconductor Optical Amplifiers Operating at 25–100° C. Proceedings of the 2018 Optical Fiber Communications Conference and Exposition (OFC).

[47] Kaizu T., Kakutani T., Akahane K., Kita T. (2020). Polarization-insensitive fiber-to-fiber gain of semiconductor optical amplifier using closely stacked InAs/GaAs quantum dots. Jpn. J. Appl. Phys..

[48] Zali A.R., Stabile R., Calabretta N. Design and Analysis of Polarization Insensitive O-Band Bulk SOA for active-passive photonic circuits. Proceedings of the CLEO: QELS_Fundamental Science.

